# Clinical Characteristics of Cutaneous Pain in Psoriasis

**DOI:** 10.3390/jcm13123610

**Published:** 2024-06-20

**Authors:** Magdalena Kotewicz, Piotr K. Krajewski, Andrzej K. Jaworek, Jacek C. Szepietowski

**Affiliations:** 1 Department of Dermatology, Venereology and Allergology, Wroclaw Medical University, 50-368 Wroclaw, Polandpiotr.krajewski@umw.edu.pl (P.K.K.); 2 Department of Dermatology, Jagiellonian University, 31-008 Kraków, Poland; andrzej.jaworek@uj.edu.pl

**Keywords:** psoriasis, pain, cutaneous pain, NRS

## Abstract

**Background:** Psoriasis is a common inflammatory disease that is often associated with itch and pain. This study aimed to evaluate the clinical characteristics of skin pain among patients with psoriasis. **Materials:** A total of 106 patients diagnosed with psoriasis were included in the study (34% female; mean age 42.1 ± 13.0 years). Disease severity was assessed using the Psoriasis Area and Severity Index (PASI). Itch severity was evaluated using the numeric rating scale (NRS) and 4-Item Itch Score (4IIS). The intensity of skin pain was measured through the NRS, short-form McGill pain questionnaire (SF-MPQ), visual analog scale (VAS), and Douleur Neuropathique-4 questionnaire (DN4). **Results:** In the past week, 84.9% of psoriasis patients reported itch, while 50% of them reported skin pain. The average NRS for itch was 4.52 ± 2.88 points, and the 4IIS yielded a mean score of 6.79 ± 4.37 points. In terms of the intensity of cutaneous pain, the mean NRS was 2.42 ± 2.96 points; the SF-MPQ score averaged 4.84 ± 7.51 points; and the VAS score was 1.92 ± 2.65 points. Furthermore, 17% of adult psoriasis patients reported neuropathic pain. In 84.9% of the participants, skin pain was concurrent with areas affected by itch, while 18.9% of patients exhibited cutaneous pain encompassing all itchy areas. The pain NRS demonstrated significant correlations with the SF-MPQ (r = 0.531, *p* < 0.001), VAS (r = 0.779, *p* < 0.001), itch NRS (r = 0.551, *p* < 0.001), and 4IIS (r = 0.569, *p* < 0.001). No association was found between the pain NRS and PASI or disease duration. **Conclusions:** Skin pain of mild intensity and itch of moderate intensity are prevalent symptoms in psoriasis patients. Strong correlations between skin pain and itch can be explained by the process of neurogenic inflammation.

## 1. Introduction

Pain is defined by the International Association for the Study of Pain (IASP) as “an unpleasant sensory or emotional experience associated with, or resembling that associated with, actual or potential tissue damage”. Pain can be classified as acute, which possesses survival value, or chronic, which is considered to be a disease [1]. Another classification of pain involves nociceptive (from tissue damage), neuropathic (from nerve damage), or neuroplastic (from a sensitized nervous system) [1]. However, in clinical practice, the different types of pain mechanisms overlap within and between patients, therefore numerous experts regard pain classification as a continuum [1]. Chronic pain is most prevalent in women, people from lower socioeconomic backgrounds, individuals living in rural areas, and military veterans [2].

The experience of pain is multidimensional, involving sensory, affective, and cognitive-evaluative aspects. Sensory aspects encompass the intensity, quality, location, and temporal, spatial, and thermal characteristics of the pain. Affective aspects involve feelings of tension, fear, and autonomic responses, while cognitive-evaluative aspects involve the overall severity of the pain experience [3].

Skin pain affects 11.2% of adults of the general population and 4.3% of patients with dermatological diseases, as demonstrated in a recent study involving 13,138 participants. This symptom was most prevalent in those with hand eczema (28.3%), rosacea (19.3%), psoriasis (19.1%), eczema (18.1%), and acne (15.3%) [4]. According to another large French study, skin pain was reported by 36.4% of dermatological patients, including 67.6% of patients with leg ulcers, 54.7% of patients with atopic dermatitis, 51.9% of patients with psoriasis, 50% of patients with pressure ulcers, and 20% of patients with sexually transmitted infections [5].

Psoriasis is a chronic, inflammatory disease that affects the skin, joints, and nails and causes severe psychosocial burdens in affected individuals [6]. It is a common dermatosis, with a prevalence ranging from 0.09% to 11.4% worldwide [7]. For a long time, psoriasis was regarded as a non-pruritic disease. However, numerous studies conducted in the last 30 years have clearly shown that itching is a highly prevalent psoriasis symptom, affecting most patients [8]. Similarly, in the past, psoriasis was considered a painless disease. However, over the past decade, studies have reported that the prevalence of skin pain ranges from 19.1% to 51.9% [9].

Skin pain and itch in psoriasis can be explained by the process of neurogenic inflammation, which involves complex neuro–immune interactions. Mediators released from peripheral sensory neurons directly attract and activate innate immune cells (mast cells, dendric cells) and adaptive immune cells (T lymphocytes) [10]. Additionally, IL-β and tumor necrosis factor (TNF)-α, two cytokines released by innate immune cells during inflammation, are directly sensed by nociceptors [10]. Mast cells also release algogenic and pruritogenic mediators, which initiate reciprocal communication with specific nociceptors on sensory nerve fibers. As a result, nerve fibers release inflammatory and vasoactive neuropeptides, which in turn activate mast cells in a feedback mechanism. This vicious cycle of mast cell and nociceptor activation further amplifies the inflammatory response as well as pain and itch [11]. 

Therefore, this study was designed to investigate the prevalence of cutaneous pain in psoriasis and provide detailed clinical characteristics of this symptom in the affected individuals.

## 2. Materials and Methods

### 2.1. Studied Group

Patients were recruited from private practice and the Department of Dermatology, Venerology, and Allergology of Wroclaw Medical University in Wroclaw, Poland, and the Department of Dermatology at the University Hospital in Cracow, Poland, between March 2023 and January 2024. Patients were included if they were >18, had psoriasis, and could read and write Polish. Patients were ineligible if at the time of enrollment they had psoriatic arthritis, another concurrent skin condition causing pain, a psychiatric diagnosis, or cognitive impairment hindering the completion of self-report questionnaires. The researchers designed an investigation sheet before commencing the study. This questionnaire encompassed demographic data, disease duration, frequency of flare-ups, family history of psoriasis, smoking habits, alcohol consumption, employment status, educational level, residential area (urban or rural), and marital status. Furthermore, the survey delved into clinical aspects of cutaneous pain and itch, such as the affected areas (limited to skin lesions or involving both lesions and non-lesional skin). Patients were also queried about the coexistence of pain and itch in the exact location. If they confirmed this overlap, they were tasked with specifying if pain extended to all itchy areas. Additionally, patients were prompted to identify regions where pain and itch coincided (face and neck; scalp; elbows; knees; hands and feet; lower back; others) and provide assessments of pain and itch severity. Lastly, the investigator conducted a clinical examination to assess the severity of psoriasis.

### 2.2. Disease Severity Assessment

The severity of psoriasis was determined using the Psoriasis Area and Severity Index (PASI), which ranges from 0 to 72 points. Higher scores on the scale indicate more severe disease. The PASI evaluates lesions based on characteristics such as erythema, induration, scaling, and the extent of the affected surface area [12].

### 2.3. Pain and Itch Assessment

Pain and itch intensity were assessed using a numerical rating scale (NRS). Participants in the study were required to evaluate the severity of the most intense itch and pain they experienced in the past week and during the whole disease. The NRS is a concise, one-dimensional scale for measuring symptom intensity, ranging from 0 (no itch/pain) to 10 (worst imaginable itch/pain) [13]. In order to evaluate pain, the following cut-off points for the pain NRS were established: ≤5 points-mild pain; >5–7 points-moderate pain; >7–10 points-severe pain. Regarding itch assessment, NRS ranges from 1 point to 3 points were considered mild itch; 4–6 points were considered moderate itch; 7–8 points were considered severe itch; and ≥9 points were considered very severe itch [13].

Furthermore, pain intensity was investigated using a short-form McGill pain questionnaire (SF-MPQ), a multidimensional assessment tool used to evaluate perceived pain, consisting of 15 descriptors, comprising 11 sensory words and 4 affective words. The Pain Rating Index consists of two subscales: a sensory subscale with 11 items and an affective subscale with 4 items, each rated on an intensity scale ranging from 0 (none) to 3 points (severe). Additionally, the SF-MPQ includes a horizontal visual analogue scale (VAS) for average pain assessment [14,15]. VAS ranges from 0, representing “no pain”, to 10 points, indicating “worst imaginable pain” [16,17].

In addition, neuropathic pain was determined using the Douleur Neuropathique-4 (DN4) questionnaire. This tool consists of four questions, including sensory descriptors and signs associated with bedside sensory examination. Questions I and II were derived from the patient interview (presence of burning, painful cold, electric shocks, tingling, prickling, numbness, and itch). In contrast, questions III and IV were based on a standardized clinical examination. Sensitivity to touch and pricking was evaluated using a soft brush and a Von Frey hair. The soft brush (applied with three movements) was additionally utilized to assess tactile (dynamic mechanical) allodynia. Pressure allodynia (static mechanical allodynia) was examined by applying blunt pressure with a finger at a level that did not induce pain in a healthy area. Each positive item was assigned a score of 1 point, while each negative item was given 0 points. The total score is determined by adding up the scores of the ten items, with a diagnostic threshold for neuropathic pain set at a total score of 4 out of 10 points [18].

Finally, the severity of itch was evaluated also using the 4-Item Itch Score. This instrument, previously utilized by our group in several studies concerning various forms of itch [19,20,21,22], was applied to assess the extent, severity, frequency, and sleep disturbances associated with chronic itch.

### 2.4. Statistical Analysis

Prior to analysis, all data underwent a normality check using Shapiro–Wilk tests. The minimum, maximum, average, and standard deviation values were determined for each variable. Depending on whether the data were normally distributed, the comparison of quantitative variables between two groups was carried out using either Student’s *t*-tests or Mann–Whitney U tests. Correlation assessments were conducted using Spearman and Pearson coefficients. For qualitative data, the Chi2 test was applied. Differences among more than two groups were evaluated using either ANOVA or the Kruskal–Wallis test. A 2-sided *p*-value of ≤0.05 was deemed statistically significant. The statistical analysis was carried out using IBM SPSS Statistics v. 26 (SPSS Inc., Chicago, IL, USA).

## 3. Results

### 3.1. Patient Characteristics

The study group consisted of 106 psoriatic individuals, including 36 women (34.0%) and 70 men (66.0%), with an age range of 18–72 (mean 42.07 ± 12.96) years. The mean PASI score was 10.93 ± 8.47 points. Most patients were working (76.4%), were cohabitants (57.5%), had secondary education (58.5%), and lived in urban areas (74.5%). The mean duration of the disease was 14.89 years (SD ±12.69) with a range from 1 to 55 years. Out of 106 participants, 31 patients (29.2%) were treated systemically with biologics, cyclosporine, methotrexate, or acitretin. A family history of psoriasis was reported by 50 individuals (47.2%). Forty-one (38.7%) of the research population were active smokers. There was a statistically significant difference (*p* = 0.004) between males and females, with 48.6% of males (*n* = 34) and 19.4% of females (*n* = 7) admitting to smoking. Twenty patients (18.9%) suffered from hypertension, including seventeen males (24.3%) and three females (8.3%). Eleven participants (10.4%) had diabetes, involving eight males (11.4%) and three females (8.3%). Two males (2.9%) had a tumor history (tumor more than 5 years before the study recruitment). Table 1 contains the data related to group characteristics.

### 3.2. Itch Characteristics

Overall, 90 (84.9%) adults with psoriasis reported itch in the past week, with a male predominance of 35 (97.2%) vs. 55 (78.6%); the difference was statistically significant (*p* = 0.011). Eighty-one percent of participants perceived itch limited to skin lesions. The average worst itch NRS in the past week was lower (4.52 ± 2.88 points) than the itch NRS during the whole disease, which was 7.04 ± 3.06 points. Women experienced significantly more intense itch than men (3.91 ± 2.89 points) (*p* = 0.003) in the past week (5.69 ± 2.51 points) and during the whole disease (8.42 ± 2.02 vs. 6.33 ± 3.27; *p* = 0.001). In the entire studied population, 4IIQ was 6.79 ± 4.37 points. All the data regarding itch are presented in Table 2.

### 3.3. Pain Characteristics

**Figure 1:**
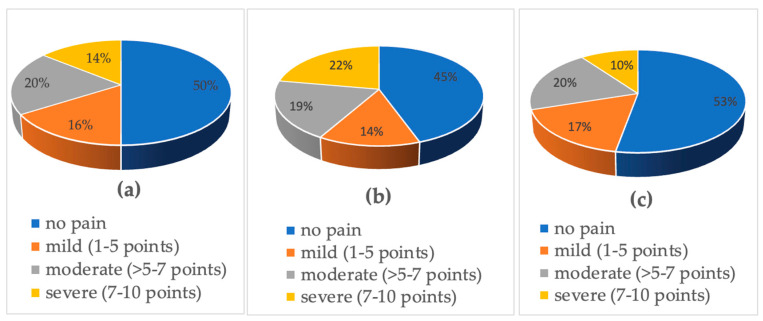
Skin pain intensity in the past week assessed with the NRS (numerical rating scale) in (**a**) the whole population, (**b**) females, and (**c**) males.

Out of 106 participants, 53 (50%) experienced pain in the past week, including 55.6% of women (*n* = 20) and 47.1% of men (*n* = 33), with an average worst pain intensity of 2.42 ± 2.96 points on the NRS scale. Moreover, cutaneous pain during the whole disease was reported by 62.3% of adults with psoriasis (*n* = 66), encompassing 63.89% of women (*n* = 23) and 61.4% of men (*n* = 43) with the mean worst pain intensity of 4.18 ± 3.837 (women 8.42 ± 2.02 vs men 4.01 ± 3.83 points); the difference was not significant. Severe skin pain was present in 14.2% of the whole population, 22.2% of females and 10% of males. In 19.8% of the whole population, 19.4% of females and 20% of males perceived moderate skin pain. Mild skin pain occurred in 16% of the whole population, 13.9% of females and 17.1% of males. The differences were not statistically significant. The data are illustrated in Figure 1. Out of 106 adults with psoriasis, 17% experienced neuropathic pain (*n* = 18), including 22.2% of women (*n* = 8) and 14.3% of men (*n* = 10). In the studied population, the SF-MPQ was 4.84 ± 7.51 points, incorporating an SF-MPQ sensory equal to 3.85 ± 6.03 points and SF-MPQ affective amounts equal to 0.99 ± 1.89 points, with no significant difference between men and women. Among all the participants, the mean VAS was 1.92 ± 2.65 points. Table 3 contains all the data concerning pain characteristics.

Pain intensity assessed with the NRS in the past week correlated significantly with the SF-MPQ total score (*r* = 0.531 *p* < 0.001), SF-MPQ sensory (*r* = 0.541 *p* < 0.001), SF-MPQ affective (*r* = 0.368 *p* = 0.007), and VAS (*r* = 0.779 *p* < 0.001). Additionally, the intensity of pain in the past week was significantly correlated with the intensity of itch (NRS) in the past week (*r* = 0.551 *p* < 0.001) as well as 4IIQ (*r* = 0.569 *p* < 0.001). However, there was no association between the intensity of pain in the past week and either the PASI or the disease duration. All the studied correlations are included in Table 4.

In the studied population, 86.8% of patients (*n* = 46) experienced pain limited to psoriatic lesions. In 84.9% (*n* = 45) of participants, pain occurred simultaneously in the areas affected by itch, while in 18.9% (*n* = 10), it affected all itchy areas. There was no statistical difference between men and women regarding these characteristics. In the whole population, pain and itch coexisted most frequently in other areas, *n* = 23 (43.4%); the lumbar area, *n* = 19 (35.8%); the knees, *n* = 18 (34.0%); and the elbows, *n* = 18 (34.0%). The distribution differed in women, with the highest prevalence of concomitant pain and itch in the lumbar area, *n* = 11 (55.0%), and scalp, *n* = 11 (55.0%). In the male population, itch and pain co-occurred in other areas, *n* = 15 (45.5%), then on the knees, *n* = 14 (42.4%), and elbows, *n* = 13 (39.4%). The prevalence of pain and itch was significantly higher in women on the face and neck (*p* = 0.005), on the scalp (*p* = 0.012), and in the lumbar area (*p* = 0.024) (Table 5).

## 4. Discussion

Itch is a very prevalent symptom in psoriasis, affecting 60–90% of patients with this disease [8]. In our study, 84.9% of the population experienced itch of moderate intensity (NRS = 4.52 ± 2.88 points), confirming these findings. We documented that the prevalence and intensity of itch were significantly higher in women than in men, which is also in line with the results of previous research [23,24].

In our study, 50% of patients with psoriasis experienced cutaneous pain in the past week, and 62.3% of our investigated population perceived skin pain during the whole disease, consistent with the foregoing research outcomes [25,26,27,28,29]. In our study, skin pain was of mild intensity as assessed with different pain assessment tools, in contrast to the results of previous investigations, which most often report skin pain of moderate intensity. For instance, Misery et al. interviewed 244 patients with psoriasis, reporting a prevalence of skin pain of 33.2% with a mean intensity of 5.83/10 points on the VAS scale [26]. In another study, García-Fernández examined 119 patients with moderate-to-severe psoriasis, in which 37.8% reported skin pain of 5.7 ± 2.6 points on the VAS scale [28] compared to the mean VAS of 1.92 ± 2.65 points in our investigation. Furthermore, Patruno et al. [25] reported skin pain in the previous week in 43.6% of psoriasis patients with a mean intensity of 7,1 points (NRS) in a studied group of 163 psoriatic individuals. Moreover, Ljosa et al. [29] recruited 139 psoriatic patients, out of whom 42.6% reported skin pain in the past 24 h with a mean intensity of 4.4 points on the NRS scale compared to the pain NRS of 2.42 ± 2.96 points in the past week and pain NRS of 4.18 ± 3.84 points during the whole disease in our study. Regarding the qualitative assessment of skin pain, the most reported qualities of skin pain reported by the patients were itchy, unpleasant, aching, sensitive, hot/burning, tender, and cramping [25,29]. 

In general practice, skin pain was more often reported by females, those with less education, those with chronic co-morbidities, and those with a more severe or longer disease duration [30]. Psoriasis patients reporting skin pain were younger than those not reporting skin pain [26], and the proportion of women with skin pain was significantly higher than the proportion of men [23,26], but we did not observe such relations in our population. Additionally, we found no association between the intensity of skin pain and the PASI, which is consistent with some studies [27,28]. However, according to some other authors, skin pain was more frequent in patients with more severe psoriasis [21,29], and improvement in the PASI predicted improvement in skin pain over time [31]. Additionally, the prevalence of skin pain was significantly higher in patients with psychiatric morbidity [23]. Regarding the clinical type of psoriasis, skin pain was reported most frequently by patients with palmoplantar and pustular psoriasis [23].

Skin pain is classically classified as nociceptive or neuropathic [32]. Nociceptive pain, which may be described as sharp, aching, and throbbing, is caused by inflammation and local tissue damage, leading to aberrant neural pathway activity [32]. In contrast, neuropathic pain is defined by IASP as “pain arising as a direct consequence of a lesion or disease affecting the somatosensory system” [33] and presents as hyperalgesia and allodynia [34]. Typical descriptors for neuropathic pain include terms such as lancinating, shooting, electrical-like, and stabbing [2]. In our cohort, neuropathic pain was present in 17% of adults with psoriasis, which is lower than in two studies conducted by Misery et al. [5,26] reporting neuropathic pain in 58.3% [5] and 63.9% [26] of psoriasis patients. However, those surveys were based only on interviews (DN4i ranged from 0 to 7 points), and the neuropathic pain cut-off score was 3 points, while in our investigation, we used the cut-off score of 4 points, and this value was obtained from questionnaires and physical examinations (DN4 ranged from 0 to 10 points) [18].

Various observations provide evidence supporting the theory of neural involvement in the development and maintenance of psoriasis, which was the subject of a recent review [35]. These observations encompass the symmetrical distribution of plaques [36], the increase in cutaneous sensory fibers and sensory neuron-derived neuropeptides in psoriatic skin [37], and the suppression of spontaneous behaviors in an imiquimod-induced mouse model of psoriasis owing to sensory denervation and the blockage of sensory neural mechanisms [38]. Azimi et al. conducted a review of cases involving changes in the presentation of pre-existing inflammatory skin diseases, such as psoriasis and AD, in patients with acquired neurological damage [39]. In 19 out of 23 cases, the resolution of skin lesions occurred in areas innervated by injured nervous tissue. Additionally, psoriatic lesions reappeared four months after a nerve injury in one patient, coinciding with the recovery of the affected nerve supply [40]. Furthermore, axotomy of cutaneous nerves in a KC-Tie2 psoriasiform model resulted in improved acanthosis and reduced numbers of CD4+ T cells and CD11c+ dermal dendric cells [41]. It was also demonstrated that TRPV1+/Nav1.8+ sensory neurons regulate the IL-17/IL-23 axis by interacting with dermal dendric cells [42]. The removal of this neuronal subset reduced the dermal dendric cell production of IL-23 and subsequently diminished IL-23-dependent IL-17 release by gd T cells, thereby reducing the inflammatory response and highlighting the regulatory role played by cutaneous sensory nerves in psoriasis [42]. 

In our study, pain and itch were limited to skin lesions in most patients. Additionally, pain and itch concurred in the same areas in the majority of individuals with psoriasis. These symptoms coexisted most frequently in areas such as the lumbar areas, knees, and elbows. The prevalence of concomitant pain and itch was significantly higher in women on the face, scalp, and lumbar area. Furthermore, we observed a strong correlation between pain and itch, suggesting that pain and itch possibly affect and enhance each other. Chronic pain and chronic itch share similar mechanisms, including peripheral and central sensitization as well as glial and immune regulation [43] and neurogenic inflammation.

Several reports have demonstrated that sensory fibers and neuropeptides, such as neurokinin A, substance P(SP), and vasoactive intestinal peptide (VIP), are increased in the psoriatic lesion compared to non-lesional skin [44,45]. Double-staining experiments have shown that the direct contacts between tryptase+ mast cells and neurofilament+ sensory nerves are more numerous in developing and mature psoriatic lesions compared to normal skin, non-lesional psoriatic skin, or lichen planus skin [46,47]. Furthermore, the contacts between tryptase+ mast cells and calcitonin gene-related peptide (CGRP)+ and SP+ nerve fibers are increased in psoriatic lesions but not the contacts between tryptase+ mast cells and VIP+ fibers [48]. This suggests an interactive role for mast cells and SP/CGRP-positive sensory nerves in the pathogenesis of psoriasis [11].

The study’s limitations include the small size of the investigated population in one country, the exclusion of children, and the fact that the patient’s subjective perceptions of pain, such as burning, tingling, and cramping, were not analyzed in this study. In addition, the significant overlap between cutaneous pain and itch in this study might be confusing, but we decided to focus on these two symptoms because they have precise definitions and quantitative methods of assessment and are commonly used in clinical trials. Moreover, the NRS measures the itch or pain within 24 h, but we decided to ask patients about what they experienced in the past week because some studies have mentioned that it is more valuable to ask patients to rate their “usual” pain over a past short period of time, e.g., one week, than to ask for “current” pain at the specific time of fulfilling a questionnaire [49]. The strength of the study is that we used a wide range of pain and itch assessment tools for the quantitative analysis, which has not been performed until now. 

## 5. Conclusions

In conclusion, skin pain and itch are prevalent symptoms in psoriasis patients. A total of 84.9% of the studied population experienced itch of moderate intensity, while 50% perceived skin pain of mild intensity. Neuropathic pain is present in 17% of individuals with psoriasis. Therefore, nociceptive pain of an inflammatory origin probably prevails in most psoriasis patients. Strong correlations between skin pain and itch can be explained by the process of neurogenic inflammation.

## Figures and Tables

**Table 1:** Demographic characteristics.

Characteristics	Whole Population (*n* = 106)	Females (*n* = 36)	Males (*n* = 70)	*p*
PASI (mean ± SD)	10.93 ± 8.47	10.18 ± 9.87	11.32 ± 7.71	NS
Age, years (mean ± SD)	42.07 ± 12.96	39.94 ± 14.00	43.16 ± 12.35	NS
Disease duration, years (mean ± SD)	14.89 ± 12.69	15.00 ± 12.23	14.83 ± 13.00	NS
Systemic treatment	31 (29.2%)	13 (36.1%)	18 (25.7%)	NS
Family history of psoriasis	50 (47.2%)	21 (58.3%)	29 (41.4%)	NS
Smokers	41 (38.7%)	7 (19.4%)	34 (48.6%)	0.004
Alcohol consumption	39 (36.8%)	10 (27.8%)	29 (41.4%)	NS
Comorbidities				
Hypertension	20 (18.9%)	3 (8.3%)	17 (24.3%)	0.04
Diabetes	11 (10.4%)	3 (8.3%)	8 (11.4%)	NS
Tumors	2 (1.9%)	0 (0%)	2 (2.9%)	NS

NS—not significant.

**Table 2:** Characteristics of itch.

	Whole Population (*n* = 106)	Females (*n* = 36)	Males (*n* = 70)	*p*
Itch in the last week	90 (84.9%)	35 (97.2%)	55 (78.6%)	0.011
Itch limited to skin lesions	86 (81%)	30 (83.3%)	56 (80.0%)	NS
NRS in the last week [points] (mean ± SD)	4.52 ± 2.88	5.69 ± 2.51	3.91 ± 2.89	0.003
NRS during the whole disease [points] (mean ± SD)	7.04 ± 3.06	8.42 ± 2.02	6.33 ± 3.27	0.001
4-Item Itch Score [points] (mean ± SD)	6.79 ± 4.37	7.69 ± 4.05	6.33 ± 4.48	NS

NRS—numerical rating scale; NS—not significant.

**Table 3:** Characteristics of skin pain.

	Whole Population (*n* = 106)	Females (*n* = 36)	Males (*n* = 70)	*p*
Pain in the past week	53 (50%)	20 (55.6%)	33 (47.1%)	NS
no pain	53 (50%)	16 (44.4%)	37 (52.9%)	NS
mild (1–5 points)	17 (16.0%)	5 (13.9%)	12 (17.1%)	NS
moderate (>5–7 points)	21 (19.8%)	7 (19.4%)	14 (20%)	NS
severe (7–10 points)	15 (14.2%)	8 (22.2%)	7 (10%)	NS
NRS in the past week [points] (mean ± SD)	2.42 ± 2.96	3.06 ± 3.30	2.09 ± 2.73	NS
Pain during the whole disease	66 (62.3%)	23 (63.89%)	43 (61.4%)	NS
NRS during the whole disease [points] (mean ± SD)	4.18 ± 3.84	8.42 ± 2.02	4.01 ± 3.83	NS
DN4 [points] (mean ± SD)	1.98 ± 1.91	2.11 ± 1.95	1.91 ± 1.90	NS
DN4 >3 points	18 (17%)	8 (22.2%)	10 (14.3%)	NS
SF-MPQ [points] (mean ± SD)	4.84 ± 7.51	5.89 ± 8.05	4.30 ± 7.22	NS
SF-MPQ sensory [points] (mean ± SD)	3.85 ± 6.03	4.39 ± 6.17	3.57 ± 5.99	NS
SF-MPQ affective [points] (mean ± SD)	0.99 ± 1.89	1.50 ± 2.24	0.73 ± 1.63	NS
VAS [points] (mean ± SD)	1.92 ± 2.65	2.32 ± 2.93	1.72 ± 2.48	NS

NRS—numerical rating scale; DN4—Douleur Neuropathique-4; SF-MPQ—short-form McGill pain questionnaire; VAS—visual analogue scale; NS—not significant.

**Table 4:** The correlation of pain intensity in the past week with the studied parameters.

	*r*	*p*
SF-MPQ Total Score	0.531	<0.001
SF-MPQ Sensory	0.541	<0.001
SF-MPQ Affective	0.368	0.007
VAS	0.779	<0.001
Itch NRS in the past week	0.551	<0.001
4IIS	0.569	<0.001
PASI		NS
Disease duration		NS

SF-MPQ—short-form McGill pain questionnaire; VAS—visual analogue scale; NRS—numerical rating scale; 4IIS—4-Item Itch Score; PASI—Psoriasis Area Severity Index; NS—not significant.

**Table 5:** The analysis of pain and itch location in the group of patients with cutaneous pain in the past week.

	Whole Population (*n* = 53)	Females (*n* = 20)	Males (*n* = 33)	*p*
Pain limited to skin lesions	46 (86.8%)	16 (80.0%)	30 (90.9%)	NS
Pain occurs in the areas affected by itch simultaneously	45 (84.9%)	18 (90.0%)	27 (81.8%)	NS
Pain is present in all areas affected by itch	10 (18.9%)	4 (20.0%)	6 (18.2%)	NS
The locations where pain and itch co-occur				
Face and neck	7 (13.2%)	6 (30.0%)	1 (3.0%)	0.005
Scalp	18 (34.0%)	11 (55.0%)	7 (21.2%)	0.012
Elbows	18 (34.0%)	5 (25.0%)	13 (39.4%)	NS
Knees	18 (34.0%)	4 (20.0%)	14 (42.4%)	NS
Hands and feet	13 (24.5%)	3 (15.0%)	10 (30.3%)	NS
Lumbar area	19 (35.8%)	11 (55.0%)	8 (24.2%)	0.024
Other areas	23 (43.4%)	8 (40.0%)	15 (45.5%)	NS

NS—not significant.

## Data Availability

The datasets analyzed or generated in the current study are available from the corresponding author upon reasonable request.
